# Risky sexual behaviour among students of a Nigerian tertiary institution

**DOI:** 10.4314/ahs.v23i4.46

**Published:** 2023-12

**Authors:** Chinedu A Idoko, Enebe O Nympha

**Affiliations:** Department of Community Medicine, College of Medicine, University of Nigeria

**Keywords:** Risky sexual behavior, students, Nigeria Tertiary Institution

## Abstract

**Background:**

While initiation of sexual activity is a part of a normal behaviour and development, it may also be associated with negative outcomes when sexual activity is initiated at too early an age, or without due attention to involved risks. These risky behaviours expose to different kinds of sexuality and reproductive health problems like STIs, HIV, unwanted and unplanned pregnancy, abortion and psychological distress.

**Objectives:**

to assess sexual risk behaviours among medical students of University of Nigeria.

**Methods:**

A descriptive cross-sectional study of sexual risk behavior of the tertiary institution students.

**Results:**

a statistical significance existed between certain socio-demographic characteristics and sexual intercourse; these characteristics are age, level of study and place of residence (p <0.05). Major reasons for sexual abstinence include ‘against my faith’, 66(37.9%), ‘waiting till marriage’, 56(32.2%), ‘not emotionally ready’, 54(31.0%) and fear of pregnancy, 10(5.7%).

**Conclusion:**

Risky sexual behaviour exist among the respondents though with varying patterns.

## Introduction

There are about 2 billion people aged 10-24 years old in the world. The youth in Nigeria account for 32% of Nigerian's 140 million people and nearly half of adolescents aged 15-19 years are sexually active. 1 This significant percentage which is enough to make an impact to the social, health and economy of the society and country at large. Human sexual behavior is any activity which could be solitary, between two persons, or in a group that induces sexual arousal. 2 It is the manner in which humans experience and express their sexuality. 3

While initiation of sexual activity is a part of a normal behaviour and development, it may also be associated with negative outcomes when sexual activity is initiated at too early an age, or without due attention to involved risks.^3^ Sexual risk behaviours include but not limited to sex without condom use, oral sex, anal sex, having multiple sexual partners, having a high-risk partner,^2^ early sexual debut, sex with a social sex worker.

Every minute, five young people worldwide become infected with HIV/AIDS.^4^ In 12 countries of sub-Saharan Africa, at least 10 percent of those aged 15 to 49 are estimated to be infected with HIV.^5^ The majority of new infections in this region are among young people aged 15 to 24. Nigeria accounted for 59% of all new HIV infections in West and Central Africa in 2016.^6^

It is known that young people often engage in high-risk behaviour like cigarette smoking, drinking of alcohol, drug use, as well as gender-based violence. These behaviours in turn lead to engagement in sexual risk behaviours.^7^ These risky behaviours expose to different kinds of sexuality and reproductive health problems like STIs, HIV, unwanted and unplanned pregnancy, abortion and psychological distress. 8,9

## Justification for the study

Sexual behavior is a complex activity affecting all aspects of human's life. The period of adolescence is characterized by periods of rapid growth, both physical and mental. Adolescents experience specific vulnerabilities that are both psychological and biological and at this period are prone to make unhealthy lifestyle choices. Among Medical Students, the demands of medical school eating into timing, psychology, social life and general indulgence of the students on all spheres indeed makes them an interesting set to study especially as regards risky sexual behaviour especially as most studies done in this respect in our environment have been mostly of the general population. Sufficing, it cannot be over-emphasized that developing valid reliable tools for assessing risky sexual behaviours and its elements especially of the study group in perspective is of paramount consideration for putting an end to increasing sexual health problems.^3^

This study aims to reflect associations of certain factors such as sociodemographic, religion, curiosity etc. to risky sexual behaviors. As a result of unsatisfactory record keeping and data available in the country, adequate data does not exist on the sexual risk behaviours of adolescents. This aim of this study is to fill this gap and encourage further study in this area.

The Research questions include but not limited to the pattern of sexual risk behaviours among medical students of University of Nigeria? factors associated with sexual risk behaviours among medical students of University of Nigeria, Enugu Campus? while the objective was to assess sexual risk behaviours among medical students of University of Nigeria, Enugu Campus with specific objectives being to identify pattern of sexual risk behaviours as well as identify factors associated with sexual risk behavior among medical students of University of Nigeria, Enugu Campus.

## Methods

### Study area

The study was carried out in the University of Nigeria, Enugu campus, Enugu state. Enugu state in the South Eastern part of Nigeria is dominated by Igbo speaking ethnic groups with other minorities

### Study design

The study was a descriptive cross-sectional study of sexual risk behaviours among medical students of University of Nigeria Enugu campus, Enugu State.

### Study population

The study population consisted of 200 level to 600 level medical students of the University of Nigeria Enugu campus, Enugu State.

### Inclusion criteria

200 level to 600 level medical students of the University of Nigeria Enugu campus who were present at the time of sampling and provided consent to participate in the study.

### Exclusion criteria

The exclusion criteria were First year medical students of the University of Nigeria who are based in Nsukka campus, non-consenting medical students and students who declined to participate in the study, medical students absent on the day of survey and acutely ill medical students who cannot participate in the study.

Sample size determination

The sample size was calculated using the statistical formula:^11^


$$\begin{array}{c} N=\left[Z^{2}P(1-P)\right]\\ D^{2} \end{array}$$

Where:

N = minimum sample size

Z = standard score at 95% confidence level which is 1.96

P = prevalence rate from previous studies which is 17.9%.^12^

D = margin of error tolerated 5%.

The sample size was calculated using the prevalence of sexual risk behavior accessed by the number sexual partners in a study done among undergraduate students in Southeast Nigeria in which a prevalence of 17.9% was obtained.


$$\begin{array}{cc} & N=(1.96{)}^{2}\times 0.179(1-0.179)/(0.05{)}^{2}\\ & =0.565/0.0025\;\;\;\;\;\;\;\;\\ & =226\;\;\;\;\;\;\;\;\;\;\;\;\; \end{array}$$

An additional 10% of the minimum sample size will be added to make for non-response.


$$\begin{array}{c} 10\text{\%}\;\text{of}\;226=(10/100)\times (226)\\ \;=22.6\\ 22.6+226=248.6\;\;\; \end{array}$$

Make room for more invalid responses and to improve accuracy, a sample size of 260 was used.

### Sampling method

The sampling method was Multi-stage Probability Sampling. Two stages of Sampling were used. These were Simple Random Sampling and Stratified Probability Sampling technique. In effect, Faculty of Medicine was chosen by Simple Random Sampling. The Faculty of Medicine was further divided into levels employing the Stratified Sampling Method of the Probability Sampling technique by dividing the faculty into strata of class levels. Medical student respondents were then randomly selected from these 5 class levels.

### Data collection

The data collection tool was a pretested, structured and self-administered questionnaire.

### Data analysis

The data was analysed using the Statistical Package for Social Science Software (SPSS) program version 25. Data was summarized using frequency tables and charts, means and standard deviations. Statistical associations were tested using Pearson's chi-square. A p-value of <0.05 was set as a criterion for establishing statistical significance. Chisquare test was used to determine the relationship among factors that affect sexual risk behaviors.

### Ethical consideration

Ethical clearance was obtained from Health Research and Ethics Committee, University of Nigeria Teaching Hospital, Ituku Ozalla, Enugu State. Participation was voluntary employing informed verbal consent. Confidentiality and anonymity of the respondent was ensured even as participants were at liberty to pull out at any point of the study at no consequence.

## Results

Table 1 shows a statistical significance with certain socio-demographic characteristics and sexual intercourse. these characteristics are age, level of study and place of residence (p <0.05).

**Table 1:** Relationship between Respondents who have/ not had sex and sociodemographic factors using cross-tabulations and chi-square

Variable	Values	Have you had sex before			Chi-square	p-value
		Yes (%)	No (%)	Total		
Age	<20 years	3 (15.0%)	17 (85.0%)	20 (100.0%)	9.534	0.023
	20-22 years	27 (25.5%)	79 (74.5%)	106 (100.0%)		
	23-25 years	31 (32.3%)	65 (67.7%)	96 (100.0%)		
	>25 years	14 (51.9%)	13 (48.1%)	27 (100.0%)		
						
Sex	Male	56 (30.8%)	126 (69.2%)	182 (100.0%)	0.135	0.713
	Female	19 (28.4%)	48 (71.6%)	67 (100.0%)		
						
Level of study	200/300 level	16 (33.3%)	32 (66.7%)	48 (100.0%)	17.802	0.000
	400 level	9 (22.0%)	32 (78.0%)	41 (100.0%)		
	500 level	27 (22.7%)	92 (77.3%)	119 (100.0%)		
	600 level	23 (56.1%)	18 (43.9%)	41 (100.0%)		
						
Place of residence	On campus with parents	3 (30.0%)	7 (70.0%)	10 (100.0%)	12.286	0.006
	On campus alone	60 (27.5%)	158 (72.5%)	218 (100.0%)		
	Off campus with parents	1 (20.0%)	4 (80.0%)	5 (100.0%)		
	Off campus alone	11 (68.8%)	5 (31.3%)	16 (100.0%)		
						

In relation to the socio-demographic factors studied, the level of study was found to be statistically significant in association with sexual risk behaviours, reporting higher sexual risk behaviours in 200/300 level students (18.8%) and 600 level students (17.1%).

Table 2 reflects major reason of abstaining from sex as ‘against my faith’, 66(37.9%), ‘waiting till marriage’, 56(32.2%), ‘not emotionally ready’, 54(31.0%) and lastly fear of pregnancy, 10(5.7%).

**Table 2:** Respondents reasons for abstaining from sex and factors which prompted sex

Variable	Values	Frequency (N= 174)	Percentage (%)
Reasons for abstaining from sexual intercourse	It's against my faith	66	37.9
	Waiting till marriage	56	32.2
	Not emotionally ready	54	31.0
	Afraid of getting pregnant	10	5.7
	Afraid of HIV/AIDS/STIs	17	9.8

Table 3, curiosity (48%) and peer Influence (32%) were the major reasons for respondent's first sexual intercourse.

**Table 3:** Respondents reasons for first sexual intercourse

Variables	Values	Frequency (N= 75)	Percentage (%)
What prompted first sexual intercourse	Peer influence	24	32.0
	Media influence	2	2.7
	Curiosity	36	48.0

Table 4 presents the frequency of various sexual risk behaviours among respondents. Majority (25) had their sexual debut between ages 18 and 19. 18.1% (45) engage in oral sex, 2.4% (6) in anal sex while 44 (17.7%) and 8 (3.2%) has had sex without contraceptive and has had sex with commercial sex workers respectively.

**Table 4:** Frequency pattern of sexual risk behaviors

Sexual Risk Behaviors (SRB)	Values	Frequency (N=249)	Percentage (%)
Early sexual debut (Age at first sexual intercourse)	<10 years	7	2.8
	10-13 years	19	7.6
	14-17 years	4	1.6
	18-19 years	25	10.0
	>19 years	20	8.0
	Never had sex	174	69.9
Multiple sexual partner (How many sexual partners have you had in the past 12 months)	One	34	13.7
	Two	21	8.4
	Above two	20	8.0
	None	174	69.9
Oral sex	Yes	45	18.1
	No	204	81.9
Anal sex	Yes	6	2.4
	No	243	97.6
Sex without contraceptive	Yes	44	17.7
	No	205	82.3
Sex with commercial sex worker	Yes	8	3.2
	No	241	96.8
High alcohol use	Yes	47	18.9
	No	202	81.1

Table 5 reflects 24(32%) of the respondents who use contraceptives all the time during sexual intercourse while 14(18.7%) of respondents had never used contraceptives.

**Table 5:** Frequency of contraceptive use amongst respondents

Variable	Values	Frequency (N=75)	Percentage (%)
How often do you use contraceptives	All the time	24	32.0
	Sometimes	21	28.0
	Occasionally	16	21.3
	Never used contraceptives	14	18.7

Table 6 presents respondents' choices on the consequences of risky sexual behaviours. 124 (49.8%) of the respondents agreed to the option of transmission of STIs and 121 (48.6%) of the respondents agreed to the option of unplanned pregnancy.

**Table 6:** Respondents view on consequences of sexual behaviors

Variable	Values	Frequency (N=249)	Percentage (%)
Consequences of sexual behaviors	Transmission of STIs	124	49.8
	Unplanned pregnancy	121	48.6
	Abortion	96	38.6
	Poor academic performance	110	44.2
	School drop out	80	32.1
	Unplanned/ Early marriage	87	34.9
	Others	31	12.4

## Discussion

The significant associations between socio-demographic characteristics and first sexual intercourse were in age, level of study and place of residence. More respondents above the age of 23 reported ever had sex compared to those below this age. First sexual intercourse was more reported by respondents in 600 level and in those who lived off campus alone. This finding may be due to the reduced restrictions experienced by these groups of respondents.

In relation to the socio-demographic factors studied, the level of study was found to be statistically significant in association with sexual risk behaviours, reporting higher sexual risk behaviours in 200/300 level students (18.8%) and 600 level students (17.1%). Other socio-demographic characteristics studied but were noted to be insignificant probably because they may have happened by chance are the student's age, sex and place of residence.

The study observed that 70% of respondents had never had sex, which was close to the findings by Ochieng who showed that the vast majority (78%) of undergraduates had not had sexual intercourse.^13^ It was observed that belief was the reason why the majority (37.9%) had not had sex emphasizing religion has a role to play in an individual's sexual behaviour.

Majority of students (48%) agreed that curiosity was the driving force for their sexual initiation. This finding is concurrent with a study carried out among school female students in Lagos which reported that the major reason for sexual initiation was curiosity.^14^ Another study carried out in Malaysia reported a similar finding.^15^

This study reports various sexual risk behaviours practiced by medical students in the university; early sexual debut, multiple sexual partners, sex with commercial workers, sex without contraceptives, oral sex and anal sex assessed. About 33% of the respondents who have had sex had their sexual debut between age 18-19, 9.3% had below the age of 10 and 25.3% had between age 10-13. This is a bit encouraging as early sexual debut is not a common risk behaviour among these students. 26.7% of the population of sexually active respondents had more than 2 sexual partners with 45.3% having had one sexual partner in the past 12 months. 18.1% of the respondents have had oral sex, 2.4% engage in anal sex and 3.2% has had sex with a commercial worker. This percentage is not in tandem with results of a study done in Southwestern Nigeria where 16.4%, 4.5% and 14% practiced oral sex, anal sex and sex with commercial workers respectively.^16^,^17^ 44% of the sexually active respondents did not use contraceptives during their last sexual intercourse. This percentage is quite large; when asked reason, majority opinionated contraceptives decreased sexual pleasure, in contrast to study done in South Eastern Nigeria on the Opinion and Use of Contraceptives among Medical Students in University of Nigeria, Enugu campus which found majority of the respondents not using contraceptives due to its unavailability.^18^

On concern of consequences of risky sexual behaviours, 49.8% and 48.6% of respondents agreed to the concern of STDS/HIV/AIDS and unwanted pregnancy respectively. 44.2% of respondents to reduced academic performance. This finding is contrary to the study done in where it was reported that all respondents, 478(100%), agreed to all the concerns such STD/HIV/AIDS, unwanted pregnancy leading to dropout from school, parental abandonment and abortion. 19, 20

## Conclusion

Risky sexual behaviour exists among the respondents though with varying patterns. Quite a number of sexually active respondents engaged in oral sex while about half of the respondents engaged in sex without contraceptives. Factors found to be associated with risky sexual behaviours include level of study, age at first sexual intercourse, substance/drug use, pornography viewing, masturbation as well as sexual abuse.

## Recommendations

This study advocates for promotion of sexual and reproductive health education among students by a diversity of avenues, including religious institutions with emphasis on the consequences of risky sexual behaviours.

## References

[1] World Bank Reproductive Health Action Plan 2013-2015.

[2] Gebhard PH, Aakanksha G Human Sexual Behaviour. Encyclopaedia Britannica.

[3] Human Sexual Activity Wikipedia.

[4] Chapin KC (2006). Molecular tests for detection of the sexually transmitted pathogens Neisseria gonorrhoeae and Chlamydia trachomatis. Med Health Rhode Island.

[5] UNICEF (2002). Young people and HIV/AIDS: Opportunity in Crisis.

[6] UNAIDS (2017). Ending AIDS: Progress towards the 90-90-90 targets.

[7] Tu X, Lou E, Gao E, Li N, Zabin LS (2012). The relationship between sexual behavior and nonsexual risk behaviors among unmarried youth in three Asian cities. Journal of Adolescent Health.

[8] Tura G, Alemseged F, Dejene S (2012). Risky sexual behavior and predisposing factors among students of Jimma University, Ethiopia. Ethiopian Journal of Health Sciences.

[9] Negeri EL (2014). Assessment of risky sexual behavior and risk perception among youth in Western Ethiopia: the influences of family and peers: A Comparative Cross-Sectional Study. BMC Public Health.

[10] Mojtaba M, Ahmadi K, Mohammad A (2016). Instruments of high-risk Sexual behaviour assessment: A Systematic Review.

[11] Dahiru T, Aliyu A, Kene TS (2006). “Statistics in Medical Research: Sample size determinatuon”. Annals of African Medicine;.

[12] Brian AJI, Umeononihu O, Echendu AD, Eke N (2016). Sexual behavior among students in a tertiary educational institution in Southeast Nigeria. Advances in Reproductive Sciences.

[13] Ochieng JA (2013). Risky sexual behavior among adolescents attending public secondary schools in Nairobi, Kenya.

[14] Odeyemi K, Onajole A, Ogunowo B (2009). Sexual behaviour and the influencing Factors among out of school female Adolescents in Mushin Market, Lagos, Nigeia. International Journal Adolescents Med. Health.

[15] Nik-Farid ND, Dahliu M, Che'Rus S, Aziz NA, Al-Sa-dat N (2015). Early Sexual initiation among Malaysian Adolescents in Welfare Institutions: A qualitative study. Arts and Social Sciences Journal.

[16] Menon AJ, Mwaba SOC, Thankian K, Lwatula C (2016). Risky Sexual Behaviour among University Students. International STD Research & Reviews.

[17] Asekun-Olarinmoye OS (2014). Effect of mass media and internet on sexual behaviour of undergraduates in Osogbo metropolis, Southwestern Nigeria. Adolescent Health Med. Ther.

[18] Idoko CA, Omotowo IB, Anyaka CU, Udo K, Ezenwosu O, Nwobi E, Ezeoke UE, Obi IE, Obienu C, Orakwue I (2018). Opinion and Use of Contraceptives among medical Students of the University of Nigeria. Africa health Sciences.

[19] Benjamin O, Nwankwo, Nwoke EA (2009). Risky Sexual Behaviours among Adolescents in Owerri Municipal: Predictors of Unmet Family Health needs. African Journal of Reproductive Health.

[20] Idoko CA, Orakwue I (2023). Factors affecting risky sexual behavior of participating adolescents of the ENSACA/ UNICEF ADOKITS Stepdown/ Rollout Program. Annals of Clinical and Biomedical Research.

